# Socio‐cognitive processing in people with eating disorders: Computerized tests of mentalizing, empathy and imitation skills

**DOI:** 10.1002/eat.23556

**Published:** 2021-05-31

**Authors:** Elisa Corsi, Valentina Cardi, Sophie Sowden, Michel‐Pierre Coll, Giammarco Cascino, Valdo Ricca, Janet Treasure, Geoffrey Bird, Alessio Maria Monteleone

**Affiliations:** ^1^ Department of Psychological Medicine, Section of Eating Disorders Institute of Psychiatry, Psychology and Neuroscience, King's College London London UK; ^2^ Psychiatry Unit, Department of Health Sciences University of Florence Florence Italy; ^3^ Department of General Psychology University of Padova Padova Italy; ^4^ School of Psychology University of Birmingham UK; ^5^ Department of Psychology McGill University Montreal Canada; ^6^ Department of Medicine, Surgery and Dentistry ‘Scuola Medica Salernitana’, Section of Neurosciences University of Salerno Salerno Italy; ^7^ Department of Experimental Psychology University of Oxford Oxford UK; ^8^ Social, Genetic and Developmental Psychiatry Centre Institute of Psychiatry, Psychology and Neuroscience, King's College London London UK; ^9^ Department of Psychiatry University of Campania “Luigi Vanvitelli” Naples Italy

**Keywords:** eating disorders, empathy, experimental tasks, imitation, mentalizing, social cognition

## Abstract

**Objective:**

Eating disorders are psychiatric illnesses characterized by extreme eating behaviors, such as sustained food restriction or loss of control over eating. Symptoms are thought to be maintained by a variety of mechanisms, one of which may be the socio‐cognitive impairments associated with eating disorders. While some previous work has addressed socio‐cognitive impairments in eating disorders, this work has relied mostly on self‐report data.

**Method:**

Here we employed computerized tests of (a) mentalizing (ability to infer the mental states of others); (b) empathy (the degree to which the emotional states of others can be identified and the degree to which the states of others impact one's own emotional state); and (c) imitation (the degree to which observation of another's actions prompts the performance of those actions); in a group of 78 women with an eating disorder and a matched control group of 66 healthy women.

**Results:**

People with eating disorders showed both hyper‐ and hypo‐mentalizing and reduced accuracy of emotional and cognitive mental state inference. They displayed less imitation of observed actions, but no differences in empathy compared to healthy controls. Although anxiety and depressive symptoms had significant effects on mentalizing, most of the observed inter‐group differences persisted.

**Discussion:**

Women with eating disorders have difficulties mentalizing and imitating observed actions despite intact non‐social automatic imitation, compared to healthy controls. These findings provide an indication that intervention modules to strengthen specific areas of social cognition might be helpful to improve patients' social skills.

## 1 INTRODUCTION

Eating disorders are psychiatric illnesses characterized by the use of extreme eating behaviors, such as sustained food restriction or loss of control over eating. They are often associated with a cascade of negative psychological and medical consequences which maintain the psychopathology by fueling numerous vicious cycles (Treasure, Duarte, & Schmidt, 2020). Treatment effectiveness is limited, and there is a great need for treatment innovation to specifically target maintaining factors of the psychopathology (Kan, Cardi, Stahl, & Treasure, 2019; Murray, Loeb, & Le Grange, 2018; Murray, Quintana, Loeb, Griffiths, & Le Grange, 2019).

One area which is receiving increasing attention because of its potential to improve the understanding and treatment of eating disorders is the socio‐cognitive abilities of individuals with eating disorders. Socio‐cognitive impairment in eating disorders have been the subject of recent systematic reviews and meta‐analyses (Monteleone, Treasure, Kan, & Cardi, 2018; Oldershaw et al., 2011) which have detailed difficulties in emotion perception and processing, a tendency to misunderstand and misinterpret signals from others, and an overestimation of the likelihood of social rejection. However, so far this area of investigation has been limited by an overreliance on self‐report data (Caglar‐Nazali et al., 2014), the lack of consensus with respect to a taxonomy of socio‐cognitive processes which may be impacted by psychopathology (Happé & Frith, 2014; Seyfarth & Cheney, 2015), and the paucity of research on the consequences of poor socio‐cognitive ability such as loneliness and isolation (c.f. Cardi, Tchanturia, & Treasure, 2018; Monteleone et al., 2019).

In this study, we investigated mentalizing, empathy and imitation—three distinct socio‐cognitive processes in which the state of another can influence the state of the self (Happé, Cook, & Bird, 2017), in individuals with eating disorders. For clarity, we define mentalizing as the ability to represent, and to accurately infer, others' mental states (Conway, Catmur, & Bird, 2019). Empathy is defined as the product of at least two separate processes: emotional identification (inference of the emotional state of the other), and affect sharing (the process by which identification of another's emotional state causes instantiation of that state in the self; Bird & Viding, 2014; Coll et al., 2017). Imitation is defined as the process by which observation of an action performed by another prompts performance of that action by the self (see Heyes, 2011). Impairments in these socio‐cognitive processes have been associated with distinct conditions; empathy with alexithymia (Bird et al., 2010); mentalizing with autism (Baron‐Cohen, Leslie, & Frith, 1985) and psychosis (Weijers et al., 2020), and atypical imitation with mirror‐touch synaesthesia (Santiesteban, Bird, Tew, Cioffi, & Banissy, 2015).

Two meta‐analyses investigated mentalizing and empathy in eating disorders and concluded that people with anorexia nervosa have difficulties in emotion identification (Leppanen, Sedgewick, Treasure, & Tchanturia, 2018) and mentalizing (Kerr‐Gaffney, Harrison, & Tchanturia, 2019a; Leppanen et al., 2018) when compared to healthy peers, but typical empathy (affect sharing; Kerr‐Gaffney et al., 2019a). No conclusions were drawn for people with bulimia nervosa (BN) given the lack of studies involving this patient group. Imitation is another process in which the state of another can influence the state of the self, but has been much less investigated in eating disorders. One study demonstrated that people with anorexia nervosa or bulimia nervosa are less accurate than healthy controls when imitating emotional facial expressions (Dapelo, Bodas, Morris, & Tchanturia, 2016). However, it is possible that these difficulties are explained by overall reduced facial expressivity (Cardi et al., 2015), rather than imitation difficulties per se.

The aim of this study was to employ computerized tasks to assess mentalizing, empathy and imitation in people with eating disorders. No previous study has simultaneously assessed these socio‐cognitive processes and their sub‐components through computerized tasks in people with eating disorders in comparison to healthy peers. Following on from previous findings (Kerr‐Gaffney et al., 2019a; Leppanen et al., 2018), we hypothesized that people with eating disorders would display greater difficulties in mentalizing and imitation compared to healthy peers, while empathy may be intact. The secondary aim was to establish whether any deficits in imitation would be associated with deficits in mentalizing and/or empathy and eating disorder symptoms. The tertiary aim was to assess the correlation between social cognition deficits and loneliness in people with eating disorders.

## 2 MATERIALS AND METHOD

### 2.1 Participants

Women (16 to 65 years‐old) with an eating disorder (i.e., anorexia nervosa or bulimia nervosa) and women with no self‐reported lifetime history of psychiatric disorders (i.e., the control group) were recruited through advertisements published on social media (i.e., Twitter, Facebook, King's College London website and newsletter), public places in south‐east London and from the eating disorder inpatient and outpatient units at South London and Maudsley NHS Hospital (patients only). Exclusion criteria included: (a) insufficient knowledge of English language, (b) visual or auditory impairment not corrected through aids, (c) diagnosis of psychosis. The eligibility screening was conducted over the phone by author EC. It included the *SCID* screening module and the *SCID‐5 for feeding and eating disorders* (First, 2016). Healthy controls answered “no” to all the questions in the SCID screening module and in the *SCID‐5 for feeding and eating disorders*, thus excluding the occurrence of past or current psychiatric disorders. The final sample consisted of 75 women with an eating disorder (38 with restrictive anorexia nervosa, 20 with binge‐purging anorexia nervosa and 17 participants with bulimia nervosa), three adolescents with restrictive anorexia nervosa and 66 healthy control women. Some data collected from the patient sample have been analyzed and published in a recent paper, with the goal of assessing the interplay between socio‐cognitive impairments, eating and affective symptoms (Monteleone et al., 2020). The recruitment method probably promoted the inclusion of young adults with a similar age to that of patients, as revealed by the Mann–Whitney test (see Table 1). Compared to healthy controls, the clinical sample had lower body mass index, fewer years of education, and reported more months of unemployment. The two groups did not differ with respect to relationship status or household composition (71.50% (*N* = 103) and 18.80% (*N* = 27) of the entire sample were single and living alone, respectively).

**TABLE 1:** Demographic and clinical characteristics of participants in the eating disorder and healthy control groups. Means (M), *SD*, Mann–Whitney test values, *p* values and effect sizes (rank‐biserial correlations) are presented

	Eating disorder group (*n* = 78) M (*SD*)	Healthy controls groups (*n* = 66) M (*SD*)	Statistic test (*W*)	*p*	Rank‐Biserial correlation
Age	26.14 (7.46)	27.43 (8.57)	2,508.5	.938	−.01
Body mass index	18.59 (4.26)	21.44 (2.41)	3,787.5	<.001	.48
Education, years	16.01 (2.90)	17.02 (2.19)	3,113	.025	.22
Months of unemployment	7.85 (22.87)	0.35 (1.71)	2,223	.021	−.13
EDE‐Q restraint eating	3.27 (1.66)	.41 (0.50)	427	<.001*	−.83
EDE‐Q eating concern	3.18 (1.44)	.17 (0.33)	183.5	<.001*	−.93
EDE‐Q shape concern	4.36 (1.71)	.73 (0.71)	326	<.001*	−.87
EDE‐Q weight concern	3.74 (1.80)	.43 (.60)	448	<.001*	−.82
SELSA‐S romantic	4.59 (1.88)	3.14 (2.01)	1,512	<.001*	−.41
SELSA‐S family	3.60 (0.88)	2.69 (0.73)	1,044	<.001*	−.59
SELSA‐S social	4.07 (1.61)	2.24 (1.12)	940.5	<.001*	−.63

Abbreviations: EDE‐Q, Eating Disorders Examination—Questionnaire; SELSA: Social and Emotional Loneliness Scale for Adults.

* Significant results following Bonferroni corrections.

This study received approval from the Research Ethics Committee of Fulham (REC approval number: 18/LO/0482). Participants provided written informed consent prior to completing the study.

### 2.2 Procedure and measures

Participants completed a demographic‐clinical questionnaire including questions about current weight and height, years of education, months of unemployment, medical diagnosis of an eating disorder and lifetime diagnosis of a comorbid psychiatric disorder. Participants also completed the following standardized self‐report questionnaires and computerized tasks in one session, on the online platforms Gorilla (https://gorilla.sc/admin/home) and Inquisit (https://www.millisecond.com ). The tasks were administered in a randomized order to avoid any potential order effects, and the testing session lasted between 60 and 90 min.

*Eating disorder symptoms*. The Eating Disorders Examination Questionnaire (EDE‐Q) (Fairburn & Beglin, 1994) consists of 36 items and measures eating disorder psychopathology over the previous 28 days. Ratings are summarized into a total score and four sub‐scales (i.e., restraint eating, eating concern, weight concern and shape concern). The Cronbach's alpha for the total score in this study was 0.91 in the patients' sample and 0.84 in the controls' sample.

*Perception of social and emotional loneliness*. The short version of the original Social and Emotional Loneliness Scale for Adults (SELSA‐S) (DiTommaso, Brannen, & Best, 2004) consists of 15 items selected from the original measure (DiTommaso & Spinner, 1993). Perceived loneliness in social, romantic and family relationships is calculated using three subscales. Cronbach's alpha for the total score in this study was 0.84.

*Depression*, *anxiety and stress symptoms*. The Depression, Anxiety and Stress Scales (DASS) (Lovibond & Lovibond, 1995) was employed to assess symptoms of depression, anxiety and stress over the previous 7 days. This scale consists of 21 items with 3 subscales (depression, anxiety and stress).

*Mentalizing*. The Movie for the Assessment of Social Cognition (MASC) (Dziobek et al., 2006) consists of a 15‐min film clip describing a real‐life scenario of social interactions between two men and two women. The actors share different levels of intimacy and experience different emotions during a party. Participants answer 45 multiple choice questions throughout the film clip, based on their understanding of the actors' mental states and emotions. Correct answers contribute to separate scores for understanding of emotional mental states (where mental state inference requires an understanding of what the characters were feeling) and cognitive mental states (where questions are based on what characters were thinking). Inaccurate answers (“errors”) are used to calculate hyper‐mentalizing (over‐interpretative or over‐elaborate mental state attribution) and hypo‐mentalizing (insufficient reasoning about others' mental states) scores.

*Empathy*. Empathy was assessed using the Empathy Accuracy Task—Revised (EAT‐R) (Coll et al., 2017; Zaki, Bolger, & Ochsner, 2008). In this task, six interviewees, defined as “Targets,” describe an emotional experience (i.e., an experience eliciting fear, anger, sadness, disgust, or happiness) and provide continuous ratings of how they feel whilst describing each experience. The six videotaped interviews are then used as stimuli for the study participants. Participants are asked to provide continuous ratings of the emotional state of the interviewee while watching the videos. The accuracy of these ratings provides a measure of emotion identification (i.e., degree of correspondence between Target's emotion and Participant's rating of Target's emotion). Coll et al. (2017) have argued that asking participants to provide ratings of their own emotions in response to each video clip provides two further measures. First, when rating of each participant's own emotional state is compared to the interviewees' emotional state, a measure of empathy as classically defined can be derived (the degree to which the Empathizer's state matches that of the Target). Second, a measure of affect sharing can be derived by calculating the degree of correspondence between each participant's ratings of their own emotional state and their ratings of the Target's emotional state. Affect sharing therefore reflects the degree to which the Empathizer's judgement of the Target's state affects the Empathizer's own state.

*Imitation*. Imitation was assessed using a task (Sowden & Shah, 2014) designed to measure automatic imitation (the tendency to automatically imitate the actions of others when not explicitly required to do so; Heyes, 2011). The task requires participants to respond with index and middle finger lifting actions in response to arbitrary cues (purple or orange squares). At the same time, participants observe an onscreen hand performing either an index or middle finger lift. Presentation of the arbitrary cue was accompanied by movement of the task‐irrelevant onscreen hand, which either performed the same action as indicated by the cue (imitatively compatible trials), or the opposite action to that required by the cue (imitatively incompatible trials). The imitative compatibility effect obtained by contrasting RTs and error rates on imitatively compatible and incompatible trials can be contrasted with the spatial compatibility effect (Simon & Rudell, 1967), which indexes the general tendency to respond faster to stimuli on the same side of space as the response. This measure is derived by contrasting RT and errors on trials when stimuli and the required response is on the same side of space (spatially compatible trials) with trials when stimuli and responses are on opposite sides of space (spatially incompatible trials).

### 2.3 Statistical analyses

The power calculation of the sample size has been reported in the supplementary material. Statistical analyses were conducted using the Statistical Package for Social Sciences, Version 22 (IBM‐SPSS Statistics 22). The normality of the data was checked through the Shapiro–Wilk test. Given that most of the data were not normally distributed, the non‐parametric Mann–Whitney and Chi‐Square tests were used to assess between‐group (clinical group versus healthy controls) differences in demographic, clinical and experimental measures. One‐way analysis of covariance (ANCOVA) was calculated to measure the effect of depression and anxiety on experimental measures. The rank biserial correlation was used to calculate effect sizes of non‐parametric comparisons: a value of 0.01 or below 0 indicates a small effect, a value of 0.15 indicates a medium effect and value of 0.25 indicates a large effect. Bonferroni corrections for multiple testing have been applied dividing 0.05 by the overall number (8) of questionnaire comparisons and task comparisons. The level of significance was set at 0.006. Given the lack of clinical and demographic differences between individuals with anorexia nervosa and bulimia nervosa (as reported in Monteleone et al., 2020), these two groups were merged into a clinical sample group based on the transdiagnostic perspective of eating disorders (Fairburn, Cooper, & Shafran, 2003).

For the MASC task, correct responses were scored as one point and incorrect responses as zero points. An overall score (total maximum score = 45) as well as four sub‐scores were calculated (i.e., accuracy of decoding emotional mental states, accuracy of decoding non‐emotional mental states, hyper‐mentalizing, and hypo‐mentalizing). Eight participants in the clinical group did not complete this task.

For the Empathic Accuracy Task‐Revised, data reduction and correlations of the time series were performed using MATLAB 7.1 (Mathworks, 2010). Average scores for each video were calculated using two second intervals and each two second average was worth one point in the subsequent time series analysis. The scores were *z*‐scored and time‐course correlations were calculated between each participant's rating of their own emotion and their rating of the Target's emotion (as a measure of Affect Sharing), each participant's rating of their own emotion and the Target's rating of their own emotion (as a measure of classical empathy), and each participant's rating of the Target's emotion and the Target's rating of their own emotion (as a measure of emotion identification). Two healthy controls and seven patients were excluded from the analysis because they did not complete some parts of the task.

For the imitation task, trials on which RT fell outside ±2.5 *SD* of the participant's mean RT were excluded from analysis (Sowden & Catmur, 2015). 2% of trials for both groups were excluded from analysis for this reason. Furthermore, for all RT analyses, trials on which an inaccurate finger‐lift response was made were discarded (7% of trials for healthy controls and 6% of trials for eating disorders). Mean RTs and error rates for each trial type (spatially and imitatively compatible, spatially compatible and imitatively incompatible, spatially incompatible and imitatively compatible, spatially and imitatively incompatible, baseline with onscreen right hand, baseline with onscreen left hand) were calculated. A mixed‐model analysis of variance (ANOVA) was carried out to calculate the main effects of Group, Spatial Compatibility and Imitative Compatibility and their interactions. Partial eta squared (*η* ^2^_*p*_) was used to calculate effect sizes: a value of 0.01 indicates a small effect, a value of 0.09 indicates a medium effect and a value of 0.25 indicates a large effect. For each individual, a spatial compatibility effect and an imitative compatibility effect were calculated (incompatible RTs‐compatible RTs in milliseconds). These scores were compared between groups using independent sample t‐tests.

Pearson's correlation analyses were performed in the patient group to investigate (a) the association between imitation and mentalizing or empathy scores, as well as eating disorder psychopathology and (b) the association between impaired mentalizing, empathy and imitation scores with perceived loneliness.

## 3 RESULTS

### 3.1 Sample characteristics

Clinical and demographic characteristics are reported in Table 1. On average, at the time of testing, patients had suffered from an eating disorder for 11.91 years (*SD* = 8.40) and had 1.78 previous hospital admissions (*SD* = 4.57; Min = 0, Max = 7). A minority were taking psychiatric medication (14 (18.1%) antidepressants, 6 (7.6%) antipsychotics); 10 (12.8%) were receiving inpatient treatment. Twenty‐three (29.4%) patients reported a current diagnosis of anxiety disorder, 26 (33.3%) reported a diagnosis of major depression, 8 (10%) had a diagnosis of obsessive–compulsive disorder and 3 (3.8%) of alcohol/substance abuse. Patients also reported higher levels of perceived loneliness in the three areas of relational life investigated (romantic, family and broad social relationships) (*p* < .001) compared to healthy controls (Table 1).

### 3.2 Mentalizing and empathy

Compared to healthy controls, patients demonstrated higher levels of both Hyper‐ and Hypo‐mentalization on the MASC (medium to large effect sizes) (Table 2). Their performance on the MASC also indicated significantly less accuracy in the identification of emotional and non‐emotional mental states (large effect sizes) (Table 2). When depression was included as covariate, the ANCOVA showed that the effect of the group (eating disorders or healthy controls) persisted on all the MASC scores except that on Hypomentalization (*F*_1,141_ = .79, *p* = .4), with a significant effect of depression on the MASC Total score (*F*_1,141_ = 4.98, *p* = .027), Accuracy of cognitive mental states (*F*_1,141_ = 5.5, *p* = .02) and Hypomentalization (*F*_1,141_ = 5.7, *p* = .018). When anxiety was included as covariate, the ANCOVA showed that the effect of the group (eating disorders or healthy controls) persisted on all the MASC scores except that on Accuracy of cognitive mental states (*F*_1,141_ = .79, *p* = .4) and Hypomentalization (*F*_1,141_ = .78, *p* = .38), with a significant effect of anxiety on these variables (*F*_1,141_ = 10.96, *p* < .01; *F*_1,141_ = 6.67, *p* = .01). A significant effect of anxiety was found also for Hypermentalization (*F*_1,141_ = 7.12, *p* < .01) and Accuracy of emotional mental states (*F*_1,141_ = 4.61, *p* = .03).

**TABLE 2:** Means (M), *SD*, Mann–Whitney tests and *p* values and effect sizes for participants' scores on the Movie for the Assessment of Social Cognition (MASC) and the Empathy Accuracy Task‐Revised (EAT‐R)

	Eating disorder sample *N*	Eating disorder sample mean (*SD*)	Healthy controls sample *N*	Healthy controlsample mean (*SD*)	Statistic test (*W*)	*p*	Rank‐Biserial correlation
MASC—total score	70	31.63 (6.15)	66	35.41 (2.91)	3,446	<.001*	0.35
MASC accuracy of emotional mental states	70	12.16 (2.60)	66	13.92 (1.63)	3,578.5	<.001*	.39
MASC accuracy of cognitive mental states	70	19.33 (4.16)	66	21.59 (2.30)	3,296.5	.003*	.29
MASC Hypermentalization score	70	5.73 (2.70)	66	4.09 (1.70)	1,604.5	<.001*	−.37
MASC Hypomentalization score	70	4.94 (3.05)	66	3.58 (1.98)	2,034	.003*	−.21
EAT‐R Emotion identification	67	.37 (.20)	64	.43 (.13)	2,735	.328	.09
EAT‐R Affect sharing	66	.27 (.20)	64	.28 (.23)	2,549	.726	.03
EAT‐R Classical empathy	66	.52 (.31)	64	.56 (.28)	2,710	.309	.10

* Significant findings following Bonferroni's correction.

In the empathy task (EAT‐R), no between‐group differences were observed in terms of identification of the Target's emotional states, affect sharing or classically‐defined empathy (small effect sizes) (Table 2).

### 3.3 Imitation

There was a significant main effect of Group, with patients (mean[ms] = 556.75; *SEM* = 11.27) responding slower than healthy controls (mean = 509.58, *SEM* = 11.16; *F*_1,103_ = 8.842, *p* = .004, *η* ^2^_*p*_ = .080). There was also a significant main effect of spatial compatibility; *F*_1,103_ = 298.69, *p* < .001, *η* ^2^_*p*_ = .74, whereby (as revealed by Bonferroni‐corrected pairwise comparisons) RTs were greater for spatially incompatible trials (mean = 555.99, *SEM* = 7.78) relative to spatially compatible trials (mean = 510.34, *SEM* = 8.27); *t*(103) = 17.28, *p* < .001, *d* = 1.67. This effect did not significantly differ between groups, as indicated by the absence of a group by spatial compatibility interaction effect (*p* = .277). There was a main effect of imitative compatibility; *F*_1,105_ = 21.93, *p* < .001, *η* ^2^_*p*_ = .18, whereby imitatively incompatible trials elicited longer RTs (mean = 539.18, SEM = 8.04) than imitatively compatible trials (mean = 527.15; SEM = 8.03); *t*(103) = 4.68, *p* < .001, *d* = 0.45. Interestingly, there was a significant interaction between group and imitative compatibility; *F*_1,105_ = 4.72, *p* = .032, *η* ^2^_*p*_ = .05, whereby imitative compatibility effects (imitatively incompatible − compatible RTs) were larger in the healthy control group (mean = 17.61, SEM = 3.71) than the clinical group (mean = 6.45, SEM = 3.55); *t*(103) = 2.17, *p* = .032, *d* = 0.42. Finally, there was a significant interaction between spatial and imitative compatibility; *F*_1,105_ = 5.18, *p* = .025, *η* ^2^_*p*_ = .05. The interaction between spatial compatibility, imitative compatibility and group was not significant (*F*_1,103_ = 0.09, *p* = .76). Due to the significant difference between groups in mean RT, analyses were rerun controlling for mean RT. The pattern of significance was unchanged. When depression and anxiety subscales were entered as covariates in the model, there were no interactions between spatial compatibility and depression or anxiety subscales (all *p* > .05) and there remained no interaction between spatial compatibility and group (eating disorders or healthy controls). No interaction was observed between anxiety and imitative compatibility (*p* > .05). Whilst there was an interaction between imitative compatibility and depression; *F*_1,101_ = 6.09, *p* = .015, *η* ^2^_*p*_ = .06, the interaction between imitative compatibility and group remained significant when controlling for depression and anxiety subscales; *F*_1,101_ = 6.43, *p* = .013, *η* ^2^_*p*_ = .06.

Analyses of the error data revealed no significant difference in overall error rates between groups (*p* = .31). There was a significant main effect of spatial compatibility; *F*_1,103_ = 111.50, *p* < .001, *η* ^2^_*p*_ = .52, whereby higher error rates were recorded during spatially incompatible (mean = 2.10, SEM = 0.14) than compatible (mean = 0.74, SEM = 0.07) trials (*t*(103) = 10.60, *p* < .001, *d* = 1.04). There was also a significant main effect of imitative compatibility (*F*_1,103_ = 23.75, *p* < .001, *η* ^2^_*p*_ = .19), with higher error rates during imitatively incompatible (mean = 1.72, SEM = 0.13) than compatible (mean = 1.12, SEM = 0.08) trials (*t*(103) = 4.86, *p* < .001, *d* = 0.47). There was not a significant interaction between group and spatial compatibility (*p* = .935), but a trend towards an interaction between group and imitative compatibility was found; *F*_1,105_ = 3.80, *p* = .054, *η* ^2^_*p*_ = .04, whereby imitative compatibility effects (imitatively incompatible – compatible error rates) were larger in the healthy control group (mean = 0.83, SEM = 0.18) than the clinical group (mean = 0.36, SEM = 0.16) group; *t*(103) = 1.95, *p* = .054, *d* = 0.38. When depression and anxiety subscales were entered as covariates in the model, there were no interactions observed between either spatial compatibility or imitative compatibility and depression or anxiety subscales (all *p* > .05), and there remained no interaction between spatial compatibility and group (eating disorders or healthy controls) when controlling for depression and anxiety. The interaction between imitative compatibility and group which was previously trending towards significance, was not significant when controlling for depression and anxiety subscales (*p* = .18).

**FIGURE 1:**
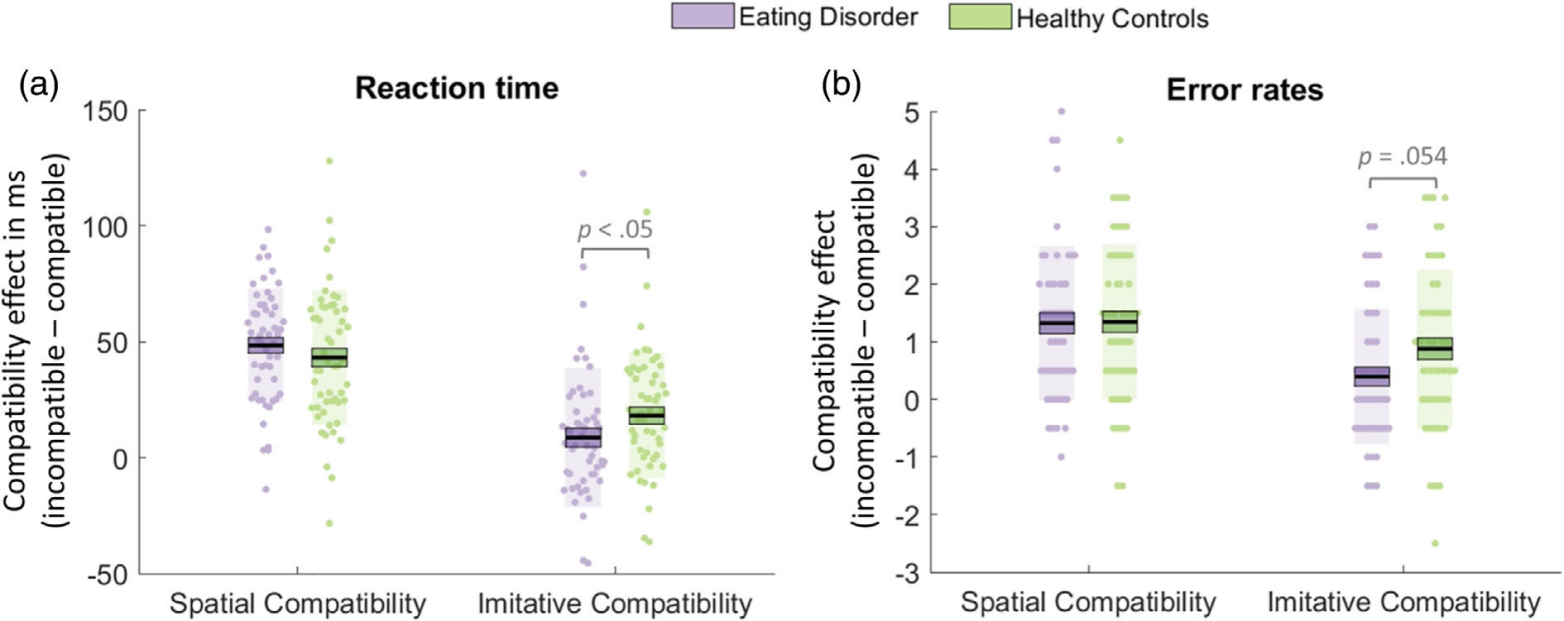
Plots of mean spatial and imitative compatibility effects as measured by reaction times (a) and error rates (b) in participants with an eating disorder (purple) and healthy controls (green). For each condition, data points indicate individual participants. The thick black horizontal line represents the sample mean, the surrounding box represents 1 *SEM* and the shaded region represents 1 *SD* [Color figure can be viewed at wileyonlinelibrary.com]

Analyses of the baseline trials revealed no significant effect of the hand presented (left or right hand) on RTs or error rates in either the healthy control or clinical groups (*p* > .05). See Figure 1 for a depiction of RT (A) and error (B) spatial and imitative compatibility effects in the healthy control and clinical groups.

### 3.4 Correlation analyses in the clinical sample

Imitative compatibility scores were not associated with empathy, mentalizing or eating disorder psychopathology (total EDE‐Q score; all *p* > .05). Difficulties identifying non‐emotional mental states (MASC) were associated with perceived loneliness within the family and more general social relationships (Table 3).

**TABLE 3:** Correlations between accuracy to identify others' mental states and emotions, imitative compatibility and perceived loneliness in the clinical sample. Confidence intervals for significant correlation coefficients are reported in brackets

	Loneliness (romantic)	Loneliness (family)	Loneliness (social)
MASC accuracy of emotional mental states	−0.05	−0.18	−0.20
MASC accuracy of cognitive mental states	−0.08	−0.32 (−0.11, −0.51)**	−0.24 (−0.02, −0.44)*
MASC hypermentalization score	−0.05	0.07	0.08
MASC hypomentalization score	0.14	0.21	0.21
Imitative compatibility effect	−0.05	0.13	0.05

* *p* < .05.

** *p* < .01.

## 4 DISCUSSION

This study investigated mentalizing, empathy and imitation in people with eating disorders and healthy peers. In comparison to healthy controls, people with eating disorders showed clear difficulties with the inference of mental states, both those requiring understanding of emotion and those that did not. Errors in mental state inference were caused by increased rates of both hyper‐ and hypo‐mentalizing. People with eating disorders also showed reduced imitation of observed actions in comparison to healthy control participants, and this was observed despite intact effects of spatial compatibility, a non‐social analogue of automatic imitation. In contrast, there was no evidence for impairments of core processes related to empathy in eating disorder patients (identifying others' emotions, affect sharing and classically‐defined empathy).

The difficulties with mental state inference observed in patients with eating disorders in this study are consistent with prior work summarized in a recent meta‐analysis (Bora & Köse, 2016) which indicated that people with eating disorders have mentalizing difficulties compared to their healthy peers. A later meta‐analysis (Leppanen et al., 2018) confirmed difficulties with mentalizing in anorexia nervosa, including when understanding of emotions is required, and difficulties understanding social interactions. Only one prior study (Brockmeyer et al., 2016) used the MASC task in individuals with anorexia nervosa, and also found reduced accuracy of emotional mental state inferences relative to healthy controls. In the present study, difficulties with mental state inference were observed both when requiring the understanding of emotional states and when not, together with evidence for both types of mentalizing errors (hyper‐ and hypo‐mentalizing). We observed that although anxiety and, partially, depression symptoms affect the mentalizing processes, the affective symptoms did not account for the difference in the mental state inference observed between patients with eating disorders and healthy controls, with the exception of hypo‐mentalizing errors. Discrepancies between the current results and those of Brockmeyer et al. (2016) could be due to the inclusion of a larger sample of individuals with eating disorders in the present work providing greater power to detect group differences, and also to the inclusion of people with bulimia nervosa (although MASC sub‐scores did not differ between people with anorexia nervosa and those with bulimia nervosa; see Monteleone et al., 2020).

With regards to empathy, no group differences were observed in terms of identifying others' emotions, affect sharing or classically‐defined empathy. These findings are only partially consistent with the literature demonstrating worse emotion recognition and intact affective empathy (affect sharing) in people with anorexia nervosa compared to healthy peers (for a review see Kerr‐Gaffney et al., 2019a). The current results extend previous data, however, as previous evidence has mostly been gathered through the use of self‐report questionnaires, and a discrepancy between self‐report and performance‐based measures of empathy has been described in other psychiatric conditions (Bonfils, Lysaker, Minor, & Salyers, 2016; Santiesteban et al., 2020).

As far as we are aware this is only the second study to assess imitation in individuals with eating disorders, and the first to assess imitation of anything other than emotional facial expressions. Assessing imitation for actions other than emotional facial expression is important, as previous work demonstrates that individuals with eating disorders pay less attention to faces and eyes than healthy controls (see Kerr‐Gaffney, Harrison, & Tchanturia, 2019b, for review), and any reduction in attention to facial stimuli may be responsible for an apparent imitation deficit using these stimuli. Results from the current study were consistent with the previous study by Dapelo et al. (2016), in that the eating disorder group showed reduced imitation. We observed that depression and anxiety did not account for the difference in imitation observed between individuals with eating disorders and healthy controls. Of note is the fact that the study conducted by Dapelo and colleagues investigated voluntary imitation in eating disorders, whereas the current study investigated automatic imitation. The consistency of results across voluntary and automatic imitation, facial and hand stimuli, in the presence of typical effects of spatial compatibility (a closely‐related but non‐social tendency for observed stimuli to affect performed actions) suggest that further investigation of imitation in eating disorders is warranted.

No relationship between imitation, mentalizing and empathy was observed, which is consistent with previous reports in autism (Spengler, Bird, & Brass, 2010), neurotypical individuals (Santiesteban, Shah, White, Bird, & Heyes, 2015) and with theoretical models (Cook, Bird, Catmur, Press, & Heyes, 2014). Thus, imitation should be considered a distinct process from mentalizing and empathy, although they likely interact in determining everyday social competence (Bird & Viding, 2014; Happé & Frith, 2014). Support for the interaction between socio‐cognitive processes and psychopathology has been provided by a previous network analysis exploring the associations between mentalizing, empathy, affective symptoms and eating disorder psychopathology in the eating disorder sample described in the current study (Monteleone et al., 2020). Findings from that study indicated that mentalizing and empathic processes interact with each other and with eating disorder and affective symptoms, and that this interplay contributes to the maintenance of eating disorder psychopathology. In the light of the present findings, it is possible to suggest that impairments in mentalizing and intact empathy processes are associated with the severity of eating disorder symptoms and internalizing symptoms in people with eating disorders (Monteleone et al., 2020). Of relevance here is the current finding that difficulties in emotional mentalizing were associated with increased loneliness. Although these findings should be considered in the light of multiple comparisons, this data is novel in the literature and supports the contribution of socio‐cognitive impairment to the poor quality of relationships perceived with friends and relatives. The lack of association between socio‐cognitive impairment and quality of intimate relationships is not surprising given the paucity or even the lack of sentimental bonding in people with eating disorders, as shown in the present study (two thirds of the sample did not hold a sentimental relationship) and in the literature (Castellini, Rossi, & Ricca, 2020).

### 4.1 Strengths and limitations

This study used well‐validated, computerized tasks to measure socio‐cognitive abilities. This is in contrast to most previous work (particularly on empathy) which utilizes self‐report measures with their inherent reliance on self‐awareness and susceptibility to bias introduced by poor self‐esteem and depression (Nosek, Hawkins, & Frazier, 2011; Roefs et al., 2011). The tasks allowed fine‐grained measurement of the different components of mentalizing and empathy, and the use of such tasks is also in line with the recent call for greater reliance on experimental procedures to clarify mechanisms maintaining eating disorder psychopathology (Glashouwer et al., 2020; Jansen, 2016).

As well as strengths, this study has also some limitations that need to be acknowledged. First, the sample size is only partially adequate for the number of variables and comparisons and the low number of people with bulimia nervosa does not allow testing for differences between eating disorder diagnostic groups. Second, patients with eating disorders were not assessed for the presence of Autism Spectrum Disorder (Westwood & Tchanturia, 2017) or alexithymia (Brewer et al., 2019), and therefore it is not possible to establish to what extent the difficulties presented would be explained by the presence of such symptoms. Third, eating disorder patients were assessed against a neurotypical standard. It is possible that rather than deficits with mentalizing or imitation per se, eating disorder patients may have specific deficits in understanding neurotypical mental states and imitating neurotypical actions. Finally, it is not possible to exclude the existence of self‐selection bias for the recruitment of participants in the control sample.

### 4.2 Conclusions

This study found that individuals with eating disorders have difficulties with mentalizing and a reduced tendency to imitate the actions of others, in the presence of intact empathic processes. These findings are based on the use of computerized and valid tasks, and corroborate and expand previous literature on socio‐cognitive impairments among this patient group. The use of tasks which can be employed in other psychiatric or neurodevelopmental disorders paves the way for transdiagnostic, dimensional investigations of the multiple facets of socio‐cognitive processes in psychopathology.

## CONFLICT OF INTEREST

The authors declare no potential conflict of interest.

## Supporting information

Supporting Information

## Data Availability

The datasets generated during and/or analysed during the current study are available from the corresponding author on reasonable request.
