# Supplementary material for: Socio‐cognitive processing in people with eating disorders: Computerized tests of mentalizing, empathy and imitation skills
Source: Int J Eat Disord. 2021 May 31;54(8):1509–18. doi: 10.1002/eat.23556 (PMC8453969; doi:10.1002/eat.23556)
Supplement: Supplementary file 1 — Supporting Information [file EAT-54-1509-s001.docx]

**Supplementary material**

*Sample size calculation*

An *a priori* power calculation was conducted based on the combined effect size for differences between individuals with anorexia nervosa and healthy controls with regards to the recognition of emotional mental states (d = 0.64) (Brockmeyer et al., 2016). The power calculation revealed that a sample size of 40 participants per group would have 80% of power to detect a medium effect size (d = 0.64) using an independent samples *t-test* with a p = 0.05 two-tailed significance level. In order to account for potential attrition, we increased the sample size to at least 60 participants per group.
